# The severity of coronary heart disease and the incidence of later diabetic retinopathy in diabetic population: A retrospective cohort study

**DOI:** 10.1371/journal.pone.0316112

**Published:** 2025-01-17

**Authors:** Ke-Hsin Ting, Po-Jen Yang, Jing-Yang Huang, Chia-Yi Lee, Shih-Chi Su, Shun-Fa Yang

**Affiliations:** 1 Division of Cardiology, Department of Internal Medicine, Changhua Christian Hospital, Yunlin, Taiwan; 2 Department of Post-Baccalaureate Medicine, College of Medicine, National Chung Hsing University, Taichung, Taiwan; 3 Department of Nursing, Hungkuang University, Taichung, Taiwan; 4 Institute of Medicine, Chung Shan Medical University, Taichung, Taiwan; 5 Department of Family and Community Medicine, Chung Shan Medical University Hospital, Taichung, Taiwan; 6 School of Medicine, Chung Shan Medical University, Taichung, Taiwan; 7 Department of Medical Research, Chung Shan Medical University Hospital, Taichung, Taiwan; 8 Department of Ophthalmology, Nobel Eye Institute, Taipei, Taiwan; 9 Whole-Genome Research Core Laboratory of Human Diseases, Chang Gung Memorial Hospital, Keelung, Taiwan; 10 Department of Medical Biotechnology and Laboratory Science, College of Medicine, Chang Gung University, Taoyuan, Taiwan; Zhejiang University, CHINA

## Abstract

**Objectives:**

The coronary heart disease (CHD) can influence the development of several diseases. The presence of CHD is correlated to a higher incidence of concurrent diabetic retinopathy (DR) in previous study. Herein, we aim to analyze the relationship between the CHD severity and following DR with different severity.

**Methods:**

A retrospective cohort study was conducted with the usage of Taiwan National Health Insurance Research Database (NHIRD). The CHD patients with DM were categorized into those with medical treatments and those received percutaneous coronary intervention (PCI) management with a 1:1 ratio. The major outcome was the development of DR, diabetic macular edema (DME) and proliferative diabetic retinopathy (PDR) 6 months after the onset of CHD.

**Results:**

There was 7317, 316, and 386 episodes of DR, DME and PDR in the mild CHD groups and 8568, 411, and 508 events of DR, DME and PDR in the severe CHD groups, respectively. The severe CHD group showed a significantly higher incidence of DR (aHR: 1.063, 95% CI: 1.038–1.089, P = 0.0324), DME (aHR: 1.412, 95% CI: 1.252–1.594, P = 0.0092) and PDR (aHR: 1.314, 95% CI: 1.172–1.473, P = 0.0113) compared to the mild CHD group. The cumulative incidence of DR was significantly higher in the severe CHD group (P < 0.001). In the subgroup analysis, the association between CHD severity and DR was more prominent in the female population (P = 0.0224).

**Conclusions:**

The severe CHD is associated with higher incidence of following DR, DME and PDR, while the incidence of DR in CHD is positively correlated to longer disease period.

## Introduction

The coronary heart disease (CHD) mainly affected the coronary artery which featured with atherosclerotic plaque formation and arterial stenosis [1]. The mild type of CHD refers to the chronic atherosclerosis which can be managed by the medical treatments such as the anti-lipid, anti-platelet and anti-adrenergic agents [2, 3]. However, the severe form of CHD including acute coronary syndrome and acute myocardial infarction usually need surgery to relief [4]. The percutaneous coronary intervention (PCI) can be applied to handle of severe CHD via physically widening the arterial cavity and the success rate of PCI was high in recent years [3, 5].

The occurrence of CHD can influence the development of several disorders [6–8]. The association between CHD and metabolic syndrome including the hypertension and diabetes mellitus (DM) had been established in the previous study [9, 10]. The existence of periodontitis may be associated with the subsequent progression of CHD [11]. Also, the Behcet’s disease with concurrent impairment of coronary artery had been reported [12, 13]. About the eye, the presence of CHD correlates to the development of open angle glaucoma [14].

The diabetic retinopathy (DR) is the vascular co-morbidity of DM which presented with vascular endothelial damage and the death of pericytes [15]. The mild form of DR can be observed regularly while the intravitreal injection (IVI) and trans pars plana vitrectomy (TPPV) may be needed in the advanced DR with diabetic macular edema (DME) and proliferative diabetic retinopathy (PDR) to rescue the visual acuity [15]. About the DR and CHD, previous researches demonstrated significantly correlation between the two diseases [16, 17]. Still, there is seldom study that discusses the influence of CHD severity on extends of following DR. Since both the CHD and DR were resulted from the vascular damage [1, 15], a relationship between them might be possible.

Accordingly, the purpose of this study is to evaluate the potential association between the severity of CHD and the degree of subsequent DR in DM population by using the data from the National Health Insurance Research Database (NHIRD) of Taiwan.

## Materials and methods

### Ethics declaration and data source

This research complied with the declaration of Helsinki in 1964 and its following amendments. This research was approved by both the National Health Insurance Administration of Taiwan and Institutional Review Board of Chung Shan Medical University Hospital (Project code: CS1-20108). The accountability of written informed consent was discarded by the above two organizations. The NHIRD has insurance data of Taiwan health insurance system and saves the medical data of above 23 million Taiwanese from January 1, 2000 to December 31, 2020. The data in Taiwan NHIRD include International Classification of Diseases Ninth Revision (ICD-9) diagnostic code, International Classification of Diseases Tenth Revision (ICD-10) diagnostic code, sex, age, economic level, education level, inhabitant place, image examination codes, laboratory examination codes, medical department codes, procedure codes, surgical codes and the international ATC codes for medicines. The data of NHIRD directly derived from the claimed codes entered by physicians, and the staffs in the National Health Insurance Bureau will confirm and transform them into electronic records.

### Patient selection

**Fig 1:**
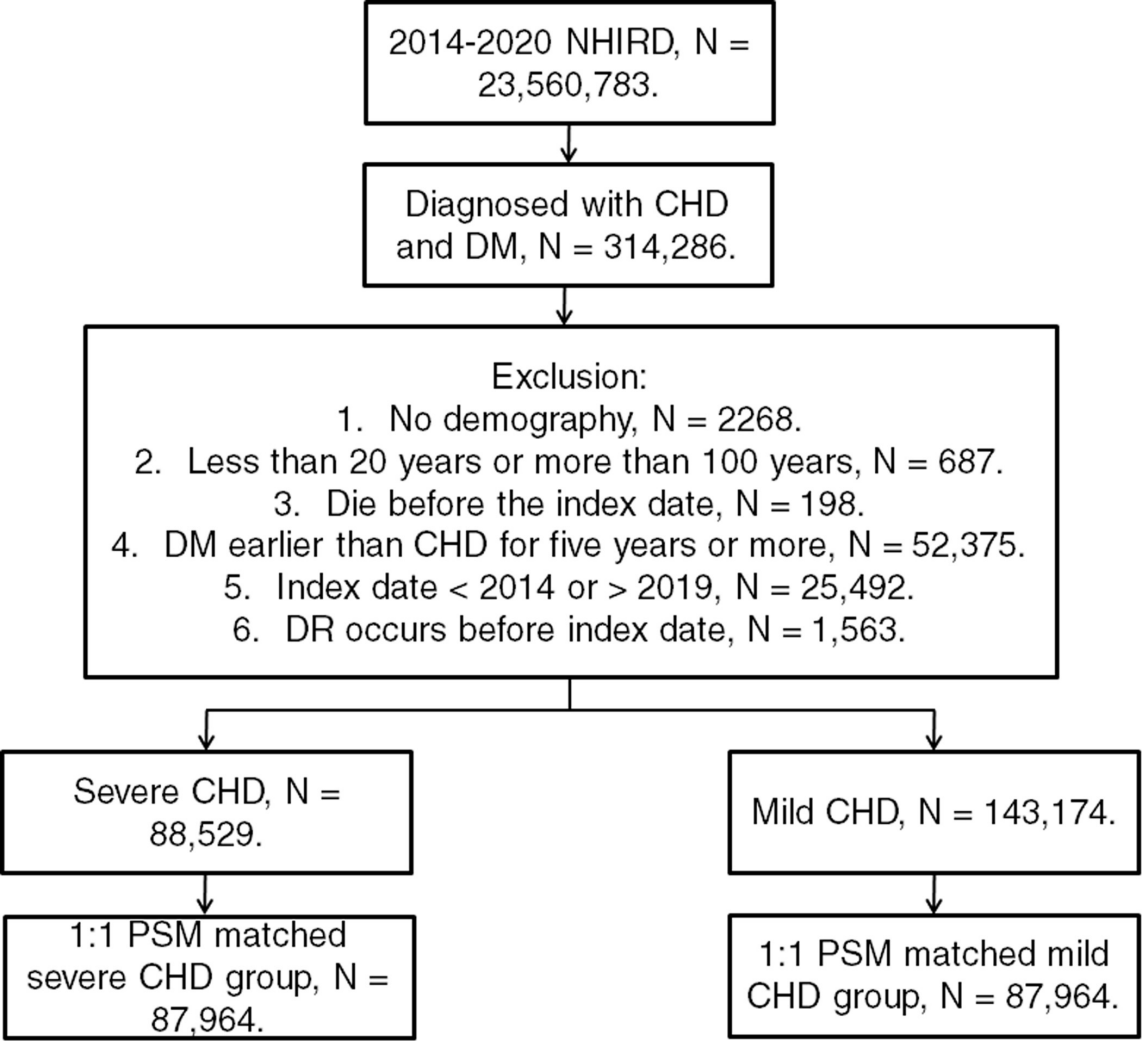
The flowchart of participant selection. NHIRD: National Health Insurance Research Database, N: number, CHD: coronary heart disease, PSM: propensity score-matching.

A retrospective population-based cohort study was conducted and patients was defined as having CHD and DM once complete the inclusion criteria: (1) the receipt of CHD diagnoses according to ICD-9 and ICD-10 codes from 2014 to 2019, (2) the receipt of DM diagnoses according to ICD-9 and ICD-10 codes from 2014 to 2019, (3) the arrangement of white blood cell differentiation count, complete blood cell count, electrocardiogram, cholesterol, triglyceride, high-density lipoprotein and low-density lipoprotein before the CHD diagnosis according to the related examination codes of Taiwan health insurance program, (4) the arrangement of blood glucose test and glycated hemoglobin exam before the DM diagnosis and (5) the patients had visited the internal physician, family medicine physician or cardiovascular specialist for at least three months. The index date of this article was set as the date 6 months after the CHD diagnosis, which means we only accounted the DRs occurred from 6 months after the CHD diagnosis. Besides, following exclusion criteria were utilized: (1) no demographics, (2) patient died before index date, (3) age less than 20-year-old or more than 100-year-old, (4) the DM diagnosis was earlier than the diagnosis of CHD for five years or more, (5) the index date was later than 2019 or earlier than 2014, and (6) the DR developed before the index date. The DR episodes developed before the index date were excluded because if we did not exclude these DR events, the time sequence between CHD and subsequent DR cannot be established which served as the main concept of this study. Also, the diabetes patients would receive routine retinal exam in Taiwan thus the rate of undiagnosed DR may be very low, although it is still possible, and the influence on the analysis may be little. To compare the influences of different CHD severities, the CHD patients were divided into the CHD individuals received medical therapy and the CHD patients received PCI treatment. One CHD case with PCI was matched to another CHD case with medical therapy via the propensity score-matching (PSM) method which adjusted the demography, systemic disorders, and related medications between the severe CHD patients received PCI and the mild CHD patients received medical therapy. Briefly, we sampled all the patients from the NHIRD who fulfilled the inclusion, then some of them were excluded according to the exclusion criteria. Finally the selected patients were categorized via the arrangement of PCI or not. After the PSM, a total number of 87964 and 87964 individuals were categorized into mild CHD group and severe CHD group. The flowchart of selection method is described in Fig 1.

### Outcome definition

The primary outcomes in this study was the DR, DME that received IVI and PDR that received TPPV which according to the following criteria: (1) the receipt of DR-related ICD-9 and ICD-10 diagnostic codes, (2) the arrangement of fundus exam, color fundus photography, optical coherence tomography or fluorescein angiography before the date of DR diagnosis according to the related examination codes, and (3) the DR diagnosis was made by an ophthalmologist. The DME and PDR was defined as severe DR in this study. For DME, the criteria include (1) the general criteria for DR, (2) the receipts of DME-related ICD-9 and ICD-10 diagnostic codes, and (3) the arrangement of IVI according to the surgery codes. About the PDR, the criteria includes (1) the general criteria for DR, (2) the receipts of PDR-related ICD-9 and ICD-10 diagnostic codes, and (3) the arrangement of IVI or TPPV according to the surgery codes, Besides, only the DR events that appeared after the index date was regarded as the outcome complement. The participants were followed till the DR occurrence, leave the National Health Insurance program (death in most cases) or the end of Taiwan NHIRD which means the December 31, 2020.

### Demographic and systemic confounders

The demographic data and systemic disorders were enrolled in the multivariable model of this study to adjust the influence of the indicators for the DR development as possible: age, sex, urbanization level, hypertension, dyslipidemia, ischemic stroke, hemorrhage stroke, peripheral vascular disease, biguanide, dipeptidyl peptidase-4 inhibitor, sodium-glucose cotransporter-2 inhibitors, glucagon-like peptide-1 agonists and statin. The determinations of these covariates were based on the demographic, ICD-9/ICD-10 and ATC codes in the Taiwan NHIRD. To ensure that the interval of these covariates in this research existed long enough to change the possibility of DR development, only the covariates that persisted/used for more than two years before the index date were included in this study. The classification of urbanization is according to an authoritative paper of Taiwan epidemiology (https://doi.org/10.29805/JHM.200606.0001) that defines the urbanization into to seven categories (sequentially): high-urbanization township, middle-urbanization township, fresh township, general township, aging township, agriculture township and remote township.

### Statistical analysis

The SAS version 9.4 (SAS Institute Inc, Cary, NC, USA) was adopted for all statistical analyses. The descriptive analyses were applied to reveal the demographic data, systemic co-morbidities and the medications between the mild CHD and severe CHD groups, and the absolute standardized difference (ASD) was adopted to analyze distributions of these parameters between the two groups. Of note, an ASD value higher than 0.1 was set as significant difference in the current research. After the process, the Cox proportional hazard regression was done to present the adjusted hazard ratios (aHR) with 95% confidence intervals (CI) of DR, DME and PDR events between the two groups. The influence of demography, systemic co-morbidities, and medicines were adjusted in the Cox proportional hazard regression analysis. We also draw the Kaplen-Meier curve to present the cumulative probability of DR, DME and PDR between the two groups. In the subgroup analyses, all the CHD patients were stratified by age and sex, and the Cox proportional hazard regression was done again to evaluate the aHR and 95% CI of DR, DME and PDR among the different subgroups. Furthermore, the interaction test was adopted to evaluate the difference of DR events in the age- and sex- CHD subgroups. The statistical significance was set as P < 0.05 in this study and a P value lesser than 0.001 was demonstrated as P < 0.001 in this study.

## Results

We collected patients from 2014 to 2019 and all the patients were followed from 2014 to 2020. The basic characters of the mild CHD and severe CHD groups are presented in the Table 1. The distributions of age and sex were similar between the two groups due to the PSM process (both ASD < 0.1). About the systemic co-morbidities, the distributions of different co-morbidities also showed insignificant difference between the two groups (all ASD < 0.1). Besides, the urbanization level and medications in the two groups demonstrated identical values (all ASD < 0.1) (Table 1). The overall dropout rate (all due to death of participant) was 6.97% and 9.14% in the mild CHD and severe CHD groups, respectively. The mean follow up period with standard deviation was 4.92 ± 0.43 and 5.01 ± 0.33 in the mild CHD and severe CHD groups, respectively.

**Table 1:** The characters between the severe and mild coronary heart disease populations.

Character	Mild CHD group (N = 87964)	Severe CHD group (N = 87964)	ASD
Sex			0.0000
Male	57220 (65.05%)	57220 (65.05%)	
Female	30744 (34.95%)	30744 (34.95%)	
Age			0.0034
<40	1434 (1.63%)	2525 (2.87%)	
40–49	5463 (6.21%)	7178 (8.16%)	
50–59	16600 (18.94%)	17654 (20.07%)	
60–69	33470 (38.05%)	25879 (29.42%)	
> = 70	30997 (35.17%)	34728 (39.48%)	
Urbanization			0.0047
1	24955 (28.37%)	22210 (25.25%)	
2	26460 (30.08%)	26275 (29.87%)	
3	15218 (17.30%)	15948 (18.13%)	
≧4	21331 (24.25%)	23531 (26.75%)	
Co-morbidity			
Hypertension	63140 (71.78%)	65384 (74.33%)	0.0087
Hyperlipidemia	49233 (55.97%)	50254 (57.13%)	0.0036
Ischemic stroke	9359 (10.64%)	10283 (11.69%)	0.0060
Hemorrhage stroke	1478 (1.68%)	2164 (2.46%)	0.0051
Peripheral vascular disease	3369 (3.83%)	3914 (4.45%)	0.0072
Co-medication			
Biguanide	53253 (60.54%)	55875 (63.52%)	0.0084
Dipeptidyl peptidase-4 inhibitor	26679 (30.33%)	33303 (37.86%)	0.0165
Sodium-glucose cotransporter-2 inhibitors	3888 (4.42%)	5436 (6.18%)	0.0243
Glucagon-like peptide-1 agonists	501 (0.57%)	669 (0.76%)	0.0092
Statin	58690 (66.72%)	59965 (68.17%)	0.0028

ASD: absolute standard difference, CHD: coronary heart disease, N: number

**Fig 2:**
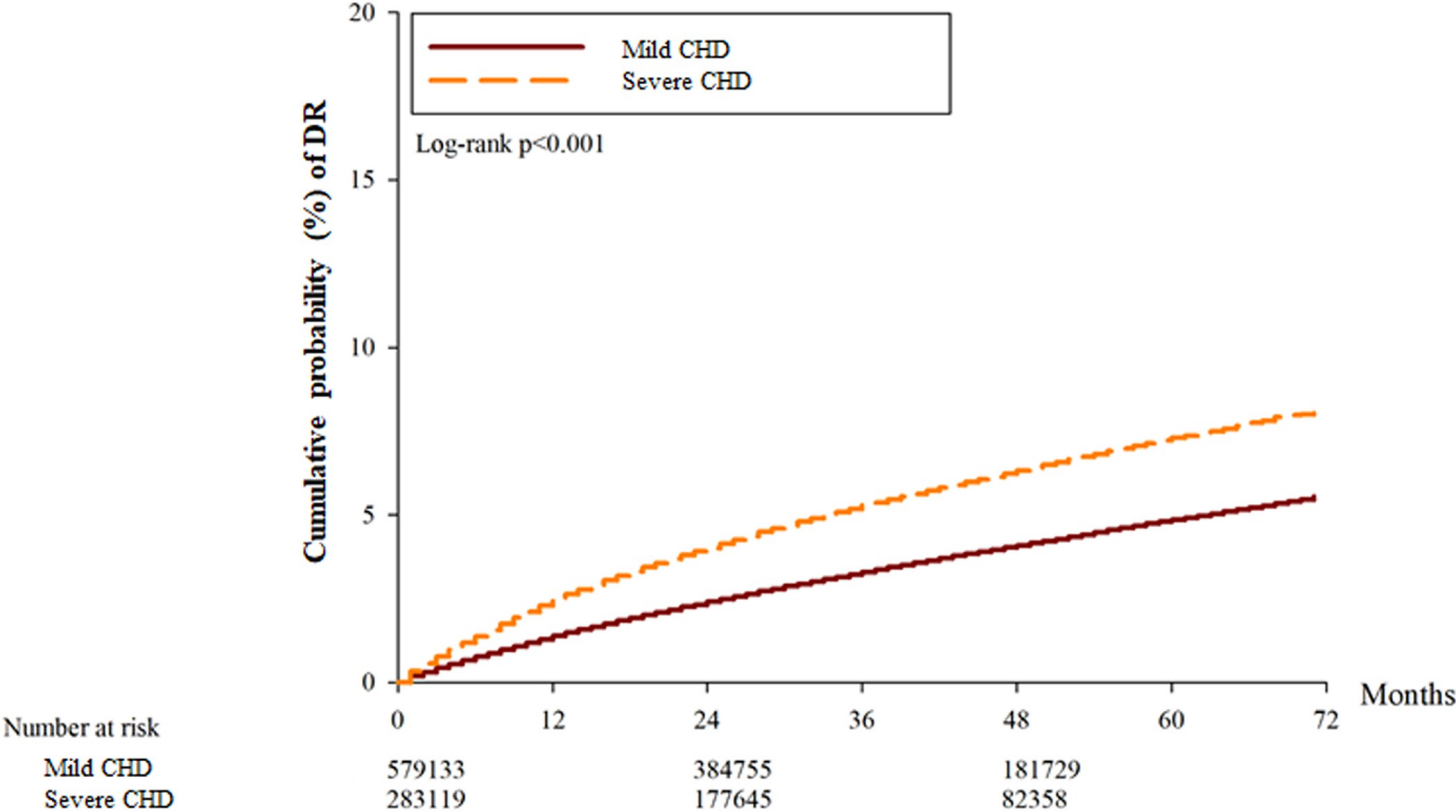
The cumulative probability of diabetic retinopathy between the two groups. CHD: coronary heart disease, DR: diabetic retinopathy.

**Fig 3:**
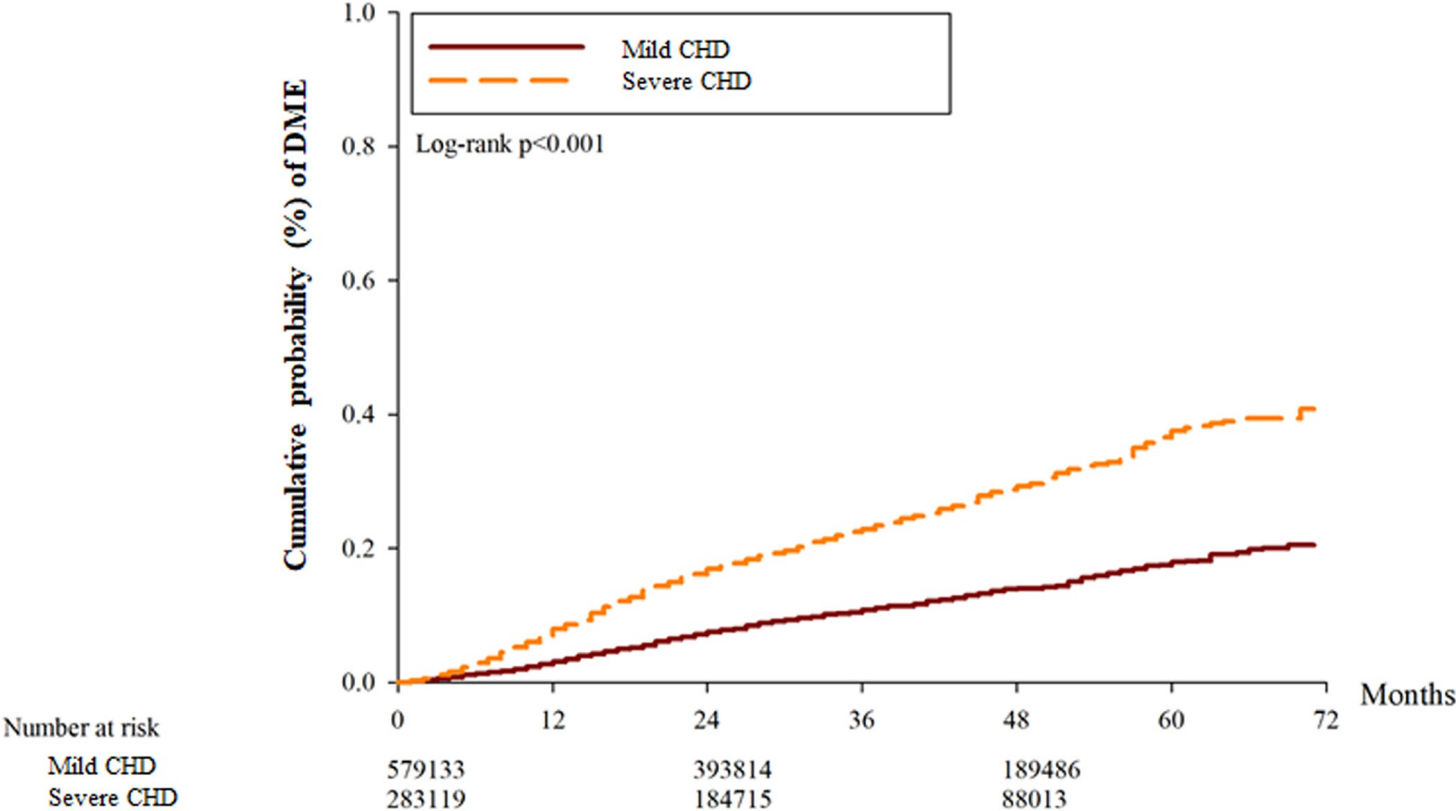
The cumulative probability of diabetic macular edema between the two groups. CHD: coronary heart disease, DME: diabetic macular edema.

**Fig 4:**
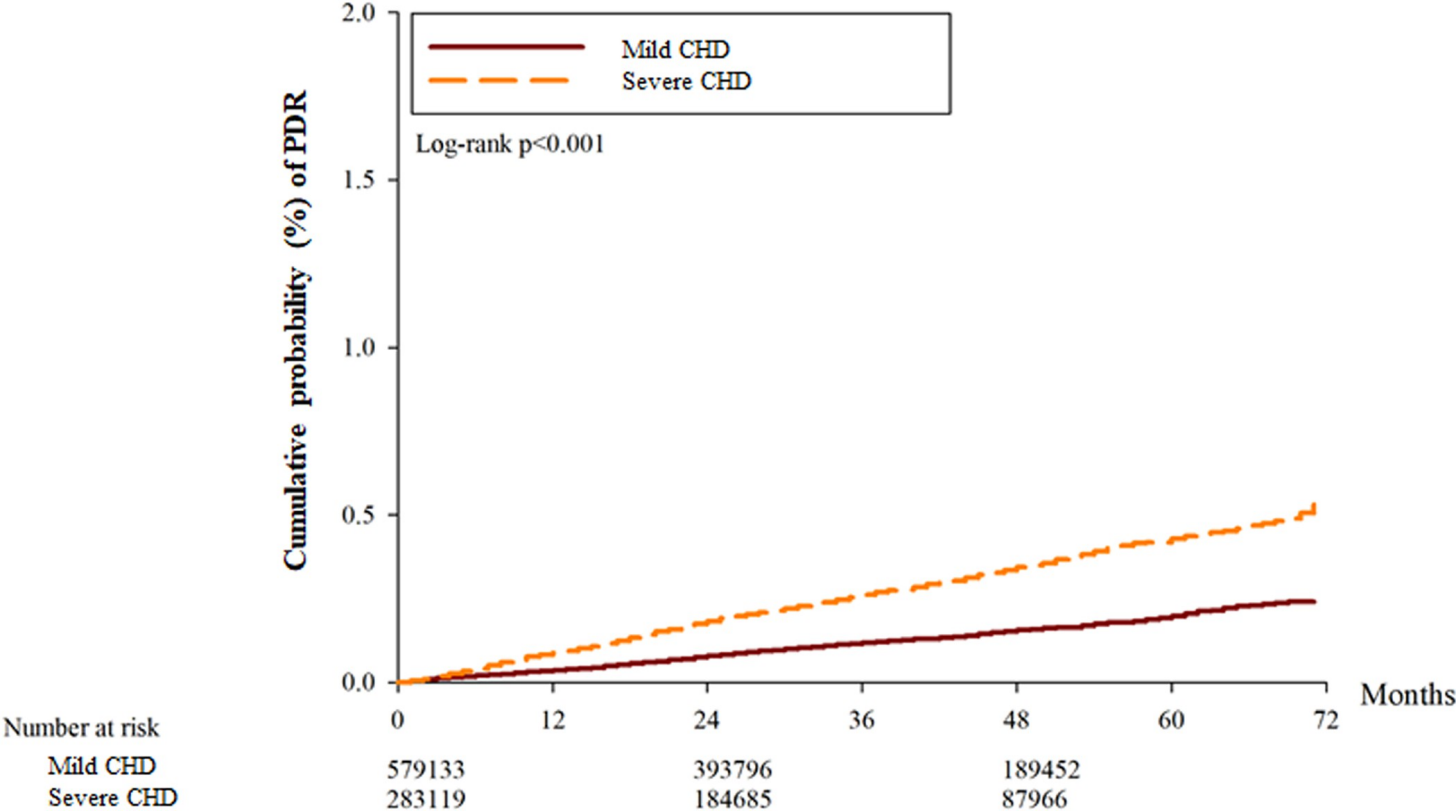
The cumulative probability of proliferative diabetic retinopathy between the two groups.

After the whole follow up period which could up to 6 years, there was 7317 and 8568 events of DR in the mild CHD and severe CHD groups, respectively. About the severe DR including those with DME and PDR, there was 316, and 386 episodes of DME and PDR in the mild CHD groups, respectively. Also, there were 411, and 508 events of DME and PDR in the severe CHD groups, respectively. The severe CHD group showed a significantly higher incidence of DR (aHR: 1.063, 95% CI: 1.038–1.089, P = 0.0324), DME (aHR: 1.412, 95% CI: 1.252–1.594, P = 0.0092) and PDR (aHR: 1.314, 95% CI: 1.172–1.473, P = 0.0113) compared to the mild CHD group according to the results of the Cox proportional hazard regression (Table 2). The cumulative incidence of DR was significantly higher in the severe CHD group (P < 0.001) (Fig 2), and the cumulative incidence of DME and PDR were also higher in the severe CHD groups (both P < 0.001) (Figs 3 and 4). Besides, the presence of hypertension did not correlate to either DR development or DR severity (both P > 0.05).

**Table 2:** The risk of diabetic retinopathy events between the two groups.

Event	Mild CHD group	Severe CHD group	P value
DR			
Person-months	7368917	7431807	
Event	7317	8568	
Crude HR (95% CI)	Reference	1.591 (1.556–1.627)	
aHR (95% CI)	Reference	1.063 (1.038–1.089)*	0.0324)*
DME			
Person-months	727083	7383526	
Event	316	411	
Crude HR (95% CI)	Reference	2.112 (1.888–2.362)	
aHR (95% CI)	Reference	1.412 (1.252–1.594))*	0.0092)*
PDR			
Person-months	724971	751556	
Event	386	508	
Crude HR (95% CI)	Reference	2.197 (1.978–2.440)	
aHR (95% CI)	Reference	1.314 (1.172–1.473))*	0.0113)*

aHR: adjusted hazard ratio, CHD: coronary heart disease, CI: confidence interval, DME: diabetic macular edema, DR: diabetic retinopathy, PDR: proliferative diabetic retinopathy

* denotes significant difference between the two groups

In the subgroup analyses, the severe CHD patients received PCI with different sex showed a significantly higher incidence of DR, DME and PDR compared to the mild CHD patients with medical treatment (all the lower limit of 95% CI > 1). About the influence of CHD severity in different sexes, the association between CHD severity and DR was more prominent in the female population (P = 0.0224) (Table 3). Concerning the subgroups stratified by age, the severe CHD subgroups demonstrated a higher incidence of DME compared to the mild CHD subgroups in all the age intervals (all the lower limit of 95% CI > 1), while the higher incidence of DR and PDR in the severe CHD subgroups compared to the mild CHD subgroups only presents in the patients younger than 70 years old (the lower limit of 95% CI > 1). The correlation of CHD severity and DR as well as PDR were more significant in the patients younger than 60 years old compared to their older counterparts (both P < 0.05) (Table 4).

**Table 3:** The subgroup analysis stratified by sex for different coronary heart disease populations.

Event	aHR	95% CI	P for interaction
DR			0.0224*
Male	1.041	1.011–1.073	
Female	1.114	1.071–1.158	
DME			0.5849
Male	1.410	1.221–1.629	
Female	1.507	1.221–1.860	
PDR			0.6148
Male	1.340	1.172–1.533	
Female	1.276	1.039–1.568	

aHR: adjusted hazard ratio, CI: confidence interval, DME: diabetic macular edema, DR: diabetic retinopathy, PDR: proliferative diabetic retinopathy

* denotes significant difference between the two groups

**Table 4:** The subgroup analysis stratified by age for different coronary heart disease populations.

Event	aHR	95% CI	P for interaction
DR			<0.0001*
<60	1.120	1.069–1.174	
60–69	1.080	1.039–1.123	
>70	1.028	0.989–1.070	
DME			0.6827
<60	1.620	1.289–2.035	
60–69	1.457	1.213–1.750	
>70	1.268	1.022–1.573	
PDR			0.0254*
<60	1.444	1.206–1.729	
60–69	1.334	1.117–1.594	
>70	1.144	0.891–1.469	

aHR: adjusted hazard ratio, CI: confidence interval, DME: diabetic macular edema, DR: diabetic retinopathy, PDR: proliferative diabetic retinopathy

* denotes significant difference between the two groups

## Discussion

The presence of CHD associates with several molecular mechanisms that could influence other diseases [7, 18, 19]. The dyslipidemia is the major pathophysiology of CHD which featured with atherosclerotic plaque and the coronary arterial stenosis [20]. The level of LDL was a well-established risk factors for the development of CHD [21], and the triglyceride is involved in the formation of atherosclerotic plaque and the subsequent CHD [21]. The inflammation is another pathway that contribute to the formation of CHD in which the cytokine including interleukin-6 and complement family were higher in the plasma of CHD patients [22]. Besides, the neutrophil-to-lymphocyte ratio can effectively predict the development of CHD [23]. The presence of CHD is also related to systemic inflammatory diseases like the inflammatory bowel disease and ankylosing spondylitis [24, 25]. In addition to the above two mechanisms, the oxidative stress was elevated significantly in CHD and can predict acute cardiovascular events [26]. On the other hand, the development of DR is associated with higher level of blood glucose which cause elevated inflammation [15]. The presence of DR and DME is related to the inflammation and oxidative stress in the retina [15], and the PDR presents with higher level of interferon-gamma compared to those patients with non-proliferative DR [27]. The lipid metabolic abnormalities in the DM patients could be a potential risk factor for DR development in previous study [28]. Because both the CHD and DR have similar pathophysiology and the vascular damage presents in both diseases [1, 15, 22], the severity of CHD may elevates the severity of DR which was supported by the findings of this study.

This study demonstrated the correlation between CHD severity and DR severity according to the existence of DME and PDR. In more details, the severe CHD increase a 17%, 30%, and 32% incidences of DR, DME and PDR than the mild CHD status. In previous study, the association of the existence of DR and higher incidence of CHD had been illustrated [29–31]. In addition, the severity of DR is also associated with the severity of concurrently developed CHD [17]. Besides, the Syntax Score that used to score complexity of CHD is correlated to the occurrence of DR in individuals with concurrent CHD and DM [32]. However, the time sequence between CHD severity and DR severity had not been investigated in these studies and the patient numbers was relatively few [17, 32]. To our knowledge, this is a preliminary study that show the correlation between advanced CHD and advanced DR. Besides, the patients with DR prior to the diagnosis of CHD was excluded from this study thus the time sequence of CHD and following DR could be established. Furthermore, the effect of age, sex and several systemic diseases including hypertension and hyperlipidemia were adjusted in the Cox proportional hazard regression, which are known risk factors for DR [15]. Besides, we excluded the DR events occurred within 6 months after the CHD diagnosis, and all the PCI managements were performed within 2 months after the diagnosis of CHD. Thus, the 4-month interval may be adequate to exclude those DR exacerbated by radio-opaque dye injection or post-PCI medications including the clopidogrel, ticagrelor and prasugrel. Consequently, the CHD severity could be an independent predisposing factor for the development and severity of DR. The possible elevation of inflammatory response and oxidative stress in the severe CHD could be the reason for the increasing incidence of severe DR. On the other hand, the cumulative incidence of DR, DME and PDR was significantly higher in the severe CHD individuals compared to those with mild CHD. In previous study, the severe CHD with is associated to higher incidence of concurrent DR [29]. In this study, we found that the longer disease interval of severe CHD could also cause higher possibility of advanced DR including the DME and PDR. Although the PCI can lead to physical stress on patients [33], the Kaplen-Meier curve analysis in this study demonstrated the development of severe DR may resulted from the persistent effect of severe CHD rather than the temporary influence of PCI.

In the subgroup analysis, the severe CHD patients with different sex illustrated a higher incidence of DR, DME and PDR compared to the mild CHD individuals. Although one research showed the higher risk of DR in the male population [34], the other studies did not report a difference of DR ratio between different sexes [35, 36]. Thus it is reasonable for the significantly higher DR/DME/PDR incidence in both sexes with severe CHD compared to the mild CHD population. Interestingly, the risk of DR between the severe CHD and mild CHD patients was more significant in the female population than men according to the interaction test. The women could experience more severe CHD compared to men in previous study [37], thus the CHD severity may be higher in the female population and cause higher chance of DR development. However, the association between severe CHD and DME as well as PDR did not show significant difference between men and women, which may implies the influence of sex on all the DR developments is not prominent. The age-subgroup analyses demonstrated an insignificant difference of DR and PDR incidences between severe CHD and mild CHD in patients older than 70 years old. Furthermore, the correlations between severe CHD and DR and PDR in the patients aged older than 70 were also lower than those with young age in interaction test. We speculate that the rate of hospitalization in patients older than 70 years old may be higher compared to the younger counterpart, thus the chance to seek ophthalmic consultation may be fewer.

In the epidemiology aspect, the CHD is a prevalent disease that influence 3–6 percent of Caucasian population [38, 39]. In the Asia population, the prevalence of CHD is estimated from 1 to 27 percent [40]. The advanced CHD with significant coronary atherosclerosis occurred in 29 percent of CHD patients despite the incidence had decreased [41], and the presence of CHD can contribute to a mortality rate of more than 40 percent in a male population with follow-up interval of 10 years [42]. On the other hand, the DR is also a prevalent disease which owns an annual incidence of 2.2 to 12.7 percent worldwide [35]. In the Chinese DM population, approximately 40 percent of patients were suffered from DR [34]. The DR and its complications including DME, PDR, tractional retinal detachment and neovascular glaucoma is the major cause of irreversible blindness in the world which could contribute to considerable economic burden [15]. Since both the CHD and DR affect a large proportion of people, the association between them which could provide a target to reduce the incidence of DR should be revealed.

There are some limitations in this study. Firstly, we applied the claimed data from the National Health Insurance Bureau for analyses which let some crucial information including the level of blood pressure, the level of blood lipid profile, the image of coronary artery, the degree of coronary artery stenosis, the details of each PCI arrangement, the post-PCI cardiovascular outcome, the level of blood glucose and glycated hemoglobin, the images of color fundus photography and optical coherence tomography, the degrees of DR and related complications, the details of IVI and TPPV, the outcome of DR-related treatment and the patient compliance of all the CHD- and DR-related medications become inaccessible. The absent of formal validation study for Taiwan NHIRD would also reduce the precision of data from Taiwan NHIRD. In addition, because the lifestyle factors were nearly not entered into the health insurance program by physician, we cannot consider them in our analysis or significant bias and inaccuracy will occur. Besides, we used the arrangement of PCI as the index for severe CHD, while the standard of PCI application could be different in each cardiologist and there may exists some people with severe CHD but refused the PCI procedure (i.e. those with extremely old age that more than 85 years) thus medical treatment was arranged in severe CHD cases. Also, the DM patients may own persistent DR before the index date which was diagnosed after the index date thus the time sequence may not be exactly credible. Finally, nearly all the participants in this study were Taiwanese, so the external validity of this study is relative low.

## Conclusion

In conclusion, the presence of severe CHD correlates to higher incidence of mild DR and severe DR including DME and PDR after adjusting several covariates. Furthermore, the DM individuals with severe CHD and older than 70 years old illustrated a lower incidence of DR compared to the younger population with severe CHD. Consequently, more aggressive CHD therapy may be suggested in the patients with concurrent CHD and DM to prevent the development of DR. Further large-scale prospective study to evaluate the influence of CHD severity on the treatment effect of DR and PDR is mandatory.

## References

[1] Sarwar N, Thompson AJ, Di Angelantonio E. Markers of inflammation and risk of coronary heart disease. Dis Markers. 2009;26(5–6):217–25. Epub 2009/09/24. doi: 10.3233/DMA-2009-0646 ; PubMed Central PMCID: PMC3833412. 19773611 PMC3833412

[2] Almeida SO, Budoff M. Effect of statins on atherosclerotic plaque. Trends Cardiovasc Med. 2019;29(8):451–5. Epub 2019/01/16. doi: 10.1016/j.tcm.2019.01.001 . 30642643

[3] Al-Lamee RK, Nowbar AN, Francis DP. Percutaneous coronary intervention for stable coronary artery disease. Heart. 2019;105(1):11–9. Epub 2018/09/23. doi: 10.1136/heartjnl-2017-312755 . 30242142

[4] Xie Q, Huang J, Zhu K, Chen Q. Percutaneous coronary intervention versus coronary artery bypass grafting in patients with coronary heart disease and type 2 diabetes mellitus: Cumulative meta-analysis. Clin Cardiol. 2021;44(7):899–906. Epub 2021/06/06. doi: 10.1002/clc.23613 ; PubMed Central PMCID: PMC8259162. 34089266 PMC8259162

[5] Hoole SP, Bambrough P. Recent advances in percutaneous coronary intervention. Heart. 2020;106(18):1380–6. Epub 2020/06/12. doi: 10.1136/heartjnl-2019-315707 . 32522821

[6] Zhong P, Li Z, Lin Y, Peng Q, Huang M, Jiang L, et al. Retinal microvasculature impairments in patients with coronary artery disease: An optical coherence tomography angiography study. Acta Ophthalmol. 2022;100(2):225–33. Epub 2021/02/26. doi: 10.1111/aos.14806 . 33629471

[7] Wirtz PH, von Känel R. Psychological Stress, Inflammation, and Coronary Heart Disease. Curr Cardiol Rep. 2017;19(11):111. Epub 2017/09/22. doi: 10.1007/s11886-017-0919-x . 28932967

[8] Wu XF, Huang JY, Chiou JY, Chen HH, Wei JC, Dong LL. Increased risk of coronary heart disease among patients with primary Sjögren’s syndrome: a nationwide population-based cohort study. Sci Rep. 2018;8(1):2209. Epub 2018/02/06. doi: 10.1038/s41598-018-19580-y ; PubMed Central PMCID: PMC5797247. 29396489 PMC5797247

[9] Escobar E. Hypertension and coronary heart disease. J Hum Hypertens. 2002;16 Suppl 1:S61–3. Epub 2002/05/03. doi: 10.1038/sj.jhh.1001345 . 11986897

[10] Rana JS, Nieuwdorp M, Jukema JW, Kastelein JJ. Cardiovascular metabolic syndrome—an interplay of, obesity, inflammation, diabetes and coronary heart disease. Diabetes Obes Metab. 2007;9(3):218–32. Epub 2007/03/30. doi: 10.1111/j.1463-1326.2006.00594.x . 17391148

[11] Priyamvara A, Dey AK, Bandyopadhyay D, Katikineni V, Zaghlol R, Basyal B, et al. Periodontal Inflammation and the Risk of Cardiovascular Disease. Curr Atheroscler Rep. 2020;22(7):28. Epub 2020/06/10. doi: 10.1007/s11883-020-00848-6 . 32514778

[12] Vural U, Kizilay M, Aglar AA. Coronary Involvement in Behçet’s Disease: what are its Risks and Prognosis? (Rare Cases and Literature Review). Braz J Cardiovasc Surg. 2019;34(6):749–58. Epub 2019/06/27. doi: 10.21470/1678-9741-2019-0003 ; PubMed Central PMCID: PMC6894034. 31241876 PMC6894034

[13] Chen H, Zhang Y, Li C, Wu W, Liu J, Zhang F, et al. Coronary involvement in patients with Behçet’s disease. Clin Rheumatol. 2019;38(10):2835–41. Epub 2019/07/06. doi: 10.1007/s10067-019-04640-z . 31273636

[14] Girkin CA, Kannel WB, Friedman DS, Weinreb RN. Glaucoma risk factor assessment and prevention: lessons from coronary heart disease. Am J Ophthalmol. 2004;138(3 Suppl):S11–8. Epub 2004/09/15. doi: 10.1016/j.ajo.2004.04.060 . 15364048

[15] Cheung N, Mitchell P, Wong TY. Diabetic retinopathy. Lancet. 2010;376(9735):124–36. Epub 2010/06/29. doi: 10.1016/S0140-6736(09)62124-3 . 20580421

[16] Cheung N, Wang JJ, Klein R, Couper DJ, Sharrett AR, Wong TY. Diabetic retinopathy and the risk of coronary heart disease: the Atherosclerosis Risk in Communities Study. Diabetes Care. 2007;30(7):1742–6. Epub 2007/03/29. doi: 10.2337/dc07-0264 . 17389333

[17] Um T, Lee DH, Kang JW, Kim EY, Yoon YH. The Degree of Diabetic Retinopathy in Patients with Type 2 Diabetes Correlates with the Presence and Severity of Coronary Heart Disease. J Korean Med Sci. 2016;31(8):1292–9. Epub 2016/08/02. doi: 10.3346/jkms.2016.31.8.1292 ; PubMed Central PMCID: PMC4951561. 27478342 PMC4951561

[18] Harrington RA. Targeting Inflammation in Coronary Artery Disease. N Engl J Med. 2017;377(12):1197–8. Epub 2017/08/29. doi: 10.1056/NEJMe1709904 . 28844177

[19] Yang J, Feng L, Ren J, Wu G, Chen S, Zhou Q, et al. Correlation between the severity of periodontitis and coronary artery stenosis in a Chinese population. Aust Dent J. 2013;58(3):333–8. Epub 2013/08/29. doi: 10.1111/adj.12087 . 23981215

[20] Kopin L, Lowenstein C. Dyslipidemia. Ann Intern Med. 2017;167(11):Itc81–itc96. Epub 2017/12/06. doi: 10.7326/AITC201712050 . 29204622

[21] Shaya GE, Leucker TM, Jones SR, Martin SS, Toth PP. Coronary heart disease risk: Low-density lipoprotein and beyond. Trends Cardiovasc Med. 2022;32(4):181–94. Epub 2021/04/20. doi: 10.1016/j.tcm.2021.04.002 . 33872757

[22] Li H, Sun K, Zhao R, Hu J, Hao Z, Wang F, et al. Inflammatory biomarkers of coronary heart disease. Front Biosci (Schol Ed). 2018;10(1):185–96. Epub 2017/09/21. doi: 10.2741/s508 . 28930526

[23] Liu Y, Ye T, Chen L, Jin T, Sheng Y, Wu G, et al. Systemic immune-inflammation index predicts the severity of coronary stenosis in patients with coronary heart disease. Coron Artery Dis. 2021;32(8):715–20. Epub 2021/04/08. doi: 10.1097/MCA.0000000000001037 . 33826540

[24] Feng KM, Chien WC, Chen YH, Sun CA, Chung CH, Chen JT, et al. Increased Risk of Acute Coronary Syndrome in Ankylosing Spondylitis Patients With Uveitis: A Population-Based Cohort Study. Front Immunol. 2022;13:890543. Epub 2022/06/28. doi: 10.3389/fimmu.2022.890543 ; PubMed Central PMCID: PMC9226308. 35757729 PMC9226308

[25] Masood F, Ehrenpreis ED, Rubin G, Russell J, Guru S, Luzzi P. State of the art review: coronary artery disease in patients with inflammatory bowel disease: mechanisms, prevalence, and outcomes. Acta Cardiol. 2022;77(4):297–306. Epub 2021/07/14. doi: 10.1080/00015385.2021.1940607 . 34254879

[26] Vassalle C, Bianchi S, Bianchi F, Landi P, Battaglia D, Carpeggiani C. Oxidative stress as a predictor of cardiovascular events in coronary artery disease patients. Clin Chem Lab Med. 2012;50(8):1463–8. Epub 2012/08/08. doi: 10.1515/cclm-2011-0919 . 22868814

[27] Ucgun NI, Zeki-Fikret C, Yildirim Z. Inflammation and diabetic retinopathy. Mol Vis. 2020;26:718–21. Epub 2020/11/20. ; PubMed Central PMCID: PMC7655973. 33209014 PMC7655973

[28] Busik JV. Lipid metabolism dysregulation in diabetic retinopathy. J Lipid Res. 2021;62:100017. Epub 2021/02/14. doi: 10.1194/jlr.TR120000981 ; PubMed Central PMCID: PMC7892987. 33581416 PMC7892987

[29] Simó R, Bañeras J, Hernández C, Rodríguez-Palomares J, Valente F, Gutierrez L, et al. Diabetic retinopathy as an independent predictor of subclinical cardiovascular disease: baseline results of the PRECISED study. BMJ Open Diabetes Res Care. 2019;7(1):e000845. Epub 2020/01/08. doi: 10.1136/bmjdrc-2019-000845 ; PubMed Central PMCID: PMC6936469. 31908800 PMC6936469

[30] Zhou JB, Zhu XR, Zhao W, Yin L, Li HB, Qi L, et al. Prediction of Proliferative Diabetic Retinopathy to Asymptomatic Obstructive Coronary Artery Disease in Chinese Type 2 Diabetes Individuals: An Exploratory Study. Metab Syndr Relat Disord. 2019;17(7):367–73. Epub 2019/05/31. doi: 10.1089/met.2018.0140 ; PubMed Central PMCID: PMC6708263. 31145036 PMC6708263

[31] Xie J, Ikram MK, Cotch MF, Klein B, Varma R, Shaw JE, et al. Association of Diabetic Macular Edema and Proliferative Diabetic Retinopathy With Cardiovascular Disease: A Systematic Review and Meta-analysis. JAMA Ophthalmol. 2017;135(6):586–93. Epub 2017/05/05. doi: 10.1001/jamaophthalmol.2017.0988 ; PubMed Central PMCID: PMC5593137. 28472362 PMC5593137

[32] Kurtul BE, Kurtul A, Yalçın F. Predictive value of the SYNTAX score for diabetic retinopathy in stable coronary artery disease patients with a concomitant type 2 diabetes mellitus. Diabetes Res Clin Pract. 2021;177:108875. Epub 2021/06/01. doi: 10.1016/j.diabres.2021.108875 . 34058301

[33] Banning AP, Baumbach A, Blackman D, Curzen N, Devadathan S, Fraser D, et al. Percutaneous coronary intervention in the UK: recommendations for good practice 2015. Heart. 2015;101 Suppl 3(Suppl 3):1–13. Epub 2015/06/05. doi: 10.1136/heartjnl-2015-307821 ; PubMed Central PMCID: PMC4484255. 26041756 PMC4484255

[34] Yin L, Zhang D, Ren Q, Su X, Sun Z. Prevalence and risk factors of diabetic retinopathy in diabetic patients: A community based cross-sectional study. Medicine (Baltimore). 2020;99(9):e19236. Epub 2020/03/03. doi: 10.1097/MD.0000000000019236 ; PubMed Central PMCID: PMC7478682. 32118727 PMC7478682

[35] Sabanayagam C, Banu R, Chee ML, Lee R, Wang YX, Tan G, et al. Incidence and progression of diabetic retinopathy: a systematic review. Lancet Diabetes Endocrinol. 2019;7(2):140–9. Epub 2018/07/15. doi: 10.1016/S2213-8587(18)30128-1 . 30005958

[36] Wat N, Wong RL, Wong IY. Associations between diabetic retinopathy and systemic risk factors. Hong Kong Med J. 2016;22(6):589–99. Epub 2016/10/26. doi: 10.12809/hkmj164869 . 27779095

[37] Khamis RY, Ammari T, Mikhail GW. Gender differences in coronary heart disease. Heart. 2016;102(14):1142–9. Epub 2016/04/30. doi: 10.1136/heartjnl-2014-306463 . 27126397

[38] Lee YH, Fang J, Schieb L, Park S, Casper M, Gillespie C. Prevalence and Trends of Coronary Heart Disease in the United States, 2011 to 2018. JAMA Cardiol. 2022;7(4):459–62. Epub 2022/01/20. doi: 10.1001/jamacardio.2021.5613 ; PubMed Central PMCID: PMC8771436. 35044425 PMC8771436

[39] Bhatnagar P, Wickramasinghe K, Wilkins E, Townsend N. Trends in the epidemiology of cardiovascular disease in the UK. Heart. 2016;102(24):1945–52. Epub 2016/08/24. doi: 10.1136/heartjnl-2016-309573 ; PubMed Central PMCID: PMC5256396. 27550425 PMC5256396

[40] Zhu KF, Wang YM, Zhu JZ, Zhou QY, Wang NF. National prevalence of coronary heart disease and its relationship with human development index: A systematic review. Eur J Prev Cardiol. 2016;23(5):530–43. Epub 2015/05/16. doi: 10.1177/2047487315587402 . 25976715

[41] Dalen JE, Alpert JS, Goldberg RJ, Weinstein RS. The epidemic of the 20(th) century: coronary heart disease. Am J Med. 2014;127(9):807–12. Epub 2014/05/09. doi: 10.1016/j.amjmed.2014.04.015 . 24811552

[42] Voutilainen A, Brester C, Kolehmainen M, Tuomainen TP. Epidemiological analysis of coronary heart disease and its main risk factors: are their associations multiplicative, additive, or interactive? Ann Med. 2022;54(1):1500–10. Epub 2022/05/24. doi: 10.1080/07853890.2022.2078875 ; PubMed Central PMCID: PMC9132387. 35603961 PMC9132387

